# Comprehensively analysis of IL33 in hepatocellular carcinoma prognosis, immune microenvironment and biological role

**DOI:** 10.1111/jcmm.18468

**Published:** 2024-06-24

**Authors:** Lifang Wei, Ping He, Zhongqiu Tan, Cheng Lin, Zhongheng Wei

**Affiliations:** ^1^ Health Management Center The Affiliated Hospital of Youjiang Medical University for Nationalities Guangxi China; ^2^ School of Laboratory Medicine Youjiang Medical University for Nationalities Guangxi China; ^3^ Department of Oncology The Affiliated Hospital of Youjiang Medical University for Nationalities Baise Guangxi China; ^4^ Guangxi Clinical Medical Research Center for Hepatobiliary Diseases The Affiliated Hospital of Youjiang Medical University for Nationalities Baise China

**Keywords:** IL33, immune, liver cancer, microenvironment, prognosis

## Abstract

IL33 plays an important role in cancer. However, the role of liver cancer remains unclear. Open‐accessed data was obtained from the Cancer Genome Atlas, Xena, and TISCH databases. Different algorithms and R packages are used to perform various analyses. Here, in our comprehensive study on IL33 in HCC, we observed its differential expression across cancers, implicating its role in cancer development. The single‐cell analysis highlighted its primary expression in endothelial cells, unveiling correlations within the HCC microenvironment. Also, the expression level of IL33 was correlated with patients survival, emphasizing its potential prognostic value. Biological enrichment analyses revealed associations with stem cell division, angiogenesis, and inflammatory response. IL33's impact on the immune microenvironment showcased correlations with diverse immune cells. Genomic features and drug sensitivity analyses provided insights into IL33's broader implications. In a pan‐cancer context, IL33 emerged as a potential tumour‐inhibitor, influencing immune‐related molecules. This study significantly advances our understanding of IL33 in cancer biology. IL33 exhibited differential expression across cancers, particularly in endothelial cells within the HCC microenvironment. IL33 is correlated with the survival of HCC patients, indicating potential prognostic value and highlighting its broader implications in cancer biology.

## 1 INTRODUCTION

Liver cancer, particularly hepatocellular carcinoma (HCC), is a common malignancy worldwide.^1^, ^2^ Its development is often associated with prolonged liver diseases, such as chronic hepatitis (especially hepatitis B and C), alcoholic liver disease or non‐alcoholic fatty liver disease.^3^ Due to the lack of distinct early symptoms, many patients are diagnosed at advanced stages, complicating treatment and adversely affecting prognosis. The treatment options for liver cancer are varied, including surgical resection, radiation therapy, chemotherapy and the emerging targeted and immunotherapies.^4^ However, the complexity and drug resistance of liver cancer often limit the effectiveness of these treatments. Consequently, researchers are seeking more effective treatment strategies to improve patient survival rates and quality of life. Moreover, early detection is crucial for successful treatment of liver cancer.^5^ Recent advances have been made in early diagnosis and treatment, such as the use of blood biomarkers and advanced imaging techniques. With a deeper understanding of the biology of liver cancer, it is hoped that more innovative treatment methods and accurate diagnostic tools will be developed in the future.

IL‐33, a key cytokine belonging to the interleukin‐1 (IL‐1) family, is widely expressed in various tissues, particularly in epithelial and endothelial cells.^6^, ^7^ It acts as a ligand for the ST2 receptor, activating a range of immune cells, including T cells, natural killer cells and dendritic cells, thereby playing a crucial role in immune responses.^8^ IL‐33 is implicated in several diseases, particularly inflammatory and autoimmune disorders like asthma, rheumatoid arthritis and allergic conditions.^9^ In the field of oncology, the role of IL‐33 has increasingly garnered attention. Studies suggest that IL‐33 may play significant roles in tumorigenesis, progression and immune evasion.^10^ For instance, in certain cancers, IL‐33 expression correlates with tumour cell proliferation, invasive capabilities and poor prognosis.^11^ Additionally, IL‐33 can modulate immune cells within the tumour microenvironment, either promoting anti‐tumour immune responses or facilitating tumour cells in evading immune surveillance.^12^ Recent studies underscore the nuanced role of IL‐33 in HCC, one of the most prevalent and lethal cancers worldwide. A notable investigation through immunohistochemistry and enzyme‐linked immunosorbent assays revealed elevated IL‐33 levels in both the serum and liver tissues of HCC patients, compared to healthy controls and adjacent non‐tumoral liver tissues.^13^ This elevation was linked not only to HCC presence but also to its metastatic progression, suggesting IL‐33's potential as a biomarker for monitoring HCC development and metastasis. The study's findings accentuate the necessity for in‐depth research into IL‐33's specific roles and mechanisms within liver cancer, potentially uncovering new avenues for therapeutic intervention or enhancing the efficacy of existing treatments. Investigating IL‐33 in the context of the liver cancer microenvironment might unveil innovative immunotherapeutic strategies or elucidate resistance mechanisms to current therapies, marking a promising frontier for exploration in cancer biology.

In our comprehensive study on IL33 in HCC, we observed its differential expression across cancers, implicating its role in cancer development. The single‐cell analysis highlighted its primary expression in endothelial cells, unveiling correlations within the HCC microenvironment. Patients with elevated IL33 in HCC exhibited better survival, emphasizing its potential prognostic value. Biological enrichment analyses revealed associations with stem cell division, angiogenesis, and inflammatory response. IL33's impact on the immune microenvironment showcased correlations with diverse immune cells. Genomic features and drug sensitivity analyses provided insights into IL33's broader implications.

## 2 METHODS

### 2.1 Public data acquisition

Our study extensively utilized public gene expression data, particularly focusing on HCC. The primary source of our data was the UCSC Xena platform, an accessible repository that aggregates large‐scale public genomic data (https://xenabrowser.net/datapages/). For HCC patients specifically, we obtained transcriptional data from The Cancer Genome Atlas (TCGA)—Liver Hepatocellular Carcinoma (LIHC) project. This dataset included transcriptome sequencing data from 50 normal liver tissue samples and 374 tumour tissue samples originating from 371 HCC patients.^14^ The expression profiles, initially available in the ‘STAR‐Counts’ format, were processed using R programming. This process involved extracting data, converting the expression values into Transcripts Per Million (TPM) units for normalization, and then further transforming these into log_2_(TPM + 1) to facilitate downstream analyses. Additionally, a human genomic reference file from the Ensemble website was employed for accurate gene symbol annotation. Before proceeding with transcriptional analyses, we executed a series of preprocessing steps to ensure data quality. These included gene symbol annotation, conversion of expression values to log_2_(TPM + 1), and implementing averaging processes for duplicate gene symbols. Clinical data associated with these samples was acquired in the format of ‘bcr‐xml’.

### 2.2 Single‐cell data of HCC

Single‐cell RNA sequencing data for HCC was sourced from the TISCH project, a platform dedicated to single‐cell transcriptomics in cancers (http://tisch.comp‐genomics.org).^15^ This high‐throughput single‐cell sequencing data offers a granular view of the cellular composition and transcriptomic landscape in HCC, providing insights into the heterogeneity within the tumour microenvironment. The single cell cohorts enrolled in this study includes GSE125449 and GSE146409.^16^, ^17^ The GSE125449 provides single‐cell transcriptome profiling of liver cancer biospecimens from nine HCC and 10 intrahepatic cholangiocarcinoma patients. The GSE146409 provides data from a study involving six patients who underwent liver resection for conditions such as colorectal metastasis, primary cholangiocarcinoma and benign liver cyst. From each patient, two types of samples were collected: malignant and non‐malignant. These samples were analysed using single‐cell RNA sequencing (scRNA‐seq) through the MARSseq technique. Cell interaction analysis was carried out by selecting specific single‐cell datasets from our study and directly analysing them to generate cell interaction maps.

### 2.3 Prognosis analysis

Prognostic assessment was carried out employing Kaplan–Meier (KM) survival curves, alongside the construction of nomograms and calibration curves for accuracy evaluation.^18^ These analyses were based on the transcriptional and clinical data, aiming to delineate the prognostic significance of IL‐33 expression levels in HCC. The KM survival curves facilitated the comparison of survival probabilities over time across different patient groups. Nomograms were developed to integrate multiple prognostic factors, including age, gender and pathological grades, offering personalized risk assessments for HCC patients. Calibration curves were subsequently used to assess the predictive accuracy of our nomogram against observed outcomes.

### 2.4 Genomic instability analysis

In the analysis of genomic and transcriptomic data, we utilized several key metrics to characterize and interpret cancer biology features and therapeutic vulnerabilities. Microsatellite instability (MSI) was assessed to understand the mutational processes affecting our samples, indicative of DNA repair pathway dysfunctions. The mRNA expression‐based stemness index (mRNAsi) and its epigenetically regulated counterpart (EREG‐mRNAsi) were calculated to quantify the degree of cellular dedifferentiation towards a stem‐like state, offering insights into tumour aggressiveness and treatment resistance. Furthermore, the tumour mutational burden (TMB) was evaluated to estimate the neoantigen load of the tumours, serving as a biomarker for the potential efficacy of immunotherapy. The MSI and TMB score was obtained from the TCGA database. The mRNAsi and EREG‐mRNAsi score was obtained from the previous study.^19^

### 2.5 Biological term analysis

Biological term analysis was conducted using Gene Set Enrichment Analysis (GSEA) analysis.^20^ GSEA is a method used for analysing microarray and high‐throughput sequencing data, primarily aimed at exploring functional enrichment patterns in gene expression data. This analysis doesn't focus on the expression changes of individual genes but evaluates the overall expression changes of predefined gene sets in specific samples. The reference pathway sets used in this study were the Kyoto Encyclopedia of Genes and Genomes (KEGG), Gene Ontology (GO) and Hallmark.

### 2.6 Immune relation analysis

Immune relation analysis was conducted using the Xcell algorithm.^21^ The Xcell algorithm is an advanced computational method designed to infer and quantify the abundance of cell types from transcriptomic data in complex tissues. This algorithm is particularly suited for analysing data from microarrays or high‐throughput sequencing technologies, aiming to identify and quantify the proportions of different cell types within tissue samples. The input for the Xcell analysis comprised gene expression datasets in the form of TPM normalized counts.

### 2.7 Drug sensitivity analysis

The data on the drug sensitivity of specific drugs was obtained from the Genomics of Drug Sensitivity in Cancer database.^22^ The GDSC database is a comprehensive resource focusing on the genomics of cancer drug sensitivity. This extensive database compiles and integrates a wealth of data regarding the responses of various cancer cell lines to a range of anti‐cancer drugs, including information on drug sensitivity, resistance mechanisms, and associated molecular markers. The IC_50_ values of specific drugs are relative quantifications derived by mapping data from the GDSC database onto the TCGA database. This approach allows us to assess the efficacy of anti‐cancer drugs in a genomic context, offering insights into potential therapeutic responses based on genetic profiles.

### 2.8 Statistical analysis

Comprehensive statistical analyses were performed using R software (version 4.0.2). We applied appropriate statistical tests based on the distribution of the data and the nature of the hypotheses being tested. This included *t*‐tests or Mann–Whitney tests for comparing continuous variables, chi‐square or Fisher's exact tests for categorical variables, and Cox proportional hazards models for survival analysis. All tests were two‐sided, and *p* < 0.05 was considered statistically significant.

## 3 RESULTS

### 3.1 Expression level of IL33 in cancer

**FIGURE 1:**
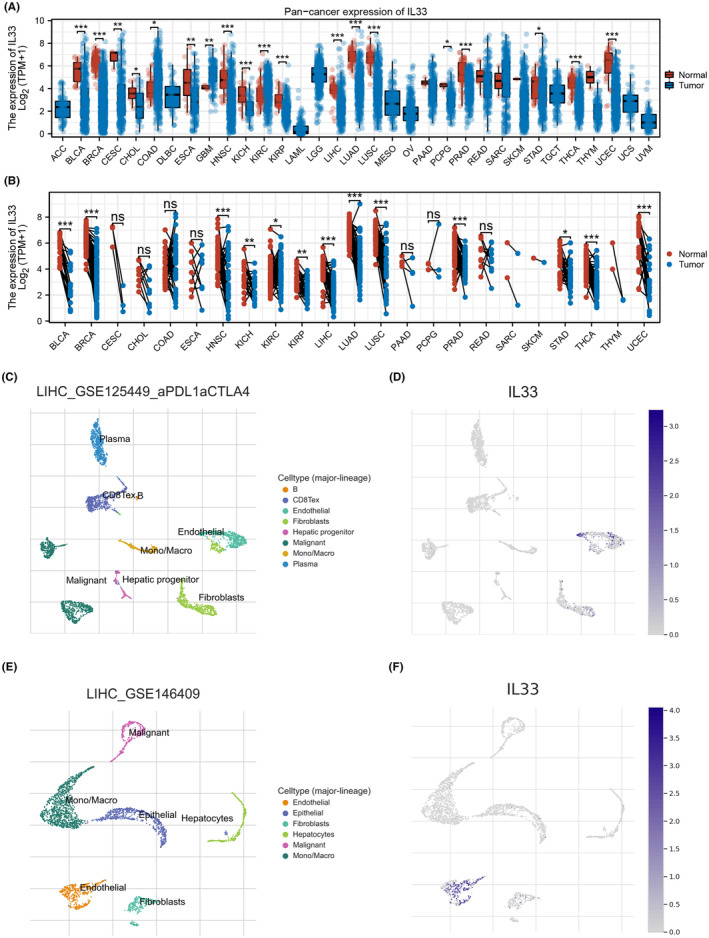
The expression pattern of IL33 in cancers. (A) Pan‐cancer analysis of IL33 expression (non‐paired), the ‘non‐paired’ analysis refers to our comparison of IL33 expression across various types of cancer without matching them to their respective normal tissue counterparts. (B) Pan‐cancer analysis of IL33 expression (paired), the ‘paired’ analysis, although not explicitly mentioned previously, involves comparing IL33 expression in cancerous tissues directly with their matched normal tissues from the same patients. (C, D) Single‐cell analysis of IL33 in HCC (GSE125449 cohort). (E, F) Single‐cell analysis of IL33 in HCC (GSE146409 cohort).

In our research, we first explored the expression patterns of IL33 in a pan‐cancer context. The findings indicated differential expression of IL33 across various cancer types, suggesting its potential role in cancer development (Figure 1A,B). Moving forward, we aimed to investigate the single‐cell expression profile of IL33, revealing its primary expression in endothelial cells (Figure 1C–F).

### 3.2 Role of endothelial cells in the HCC microenvironment

**FIGURE 2:**
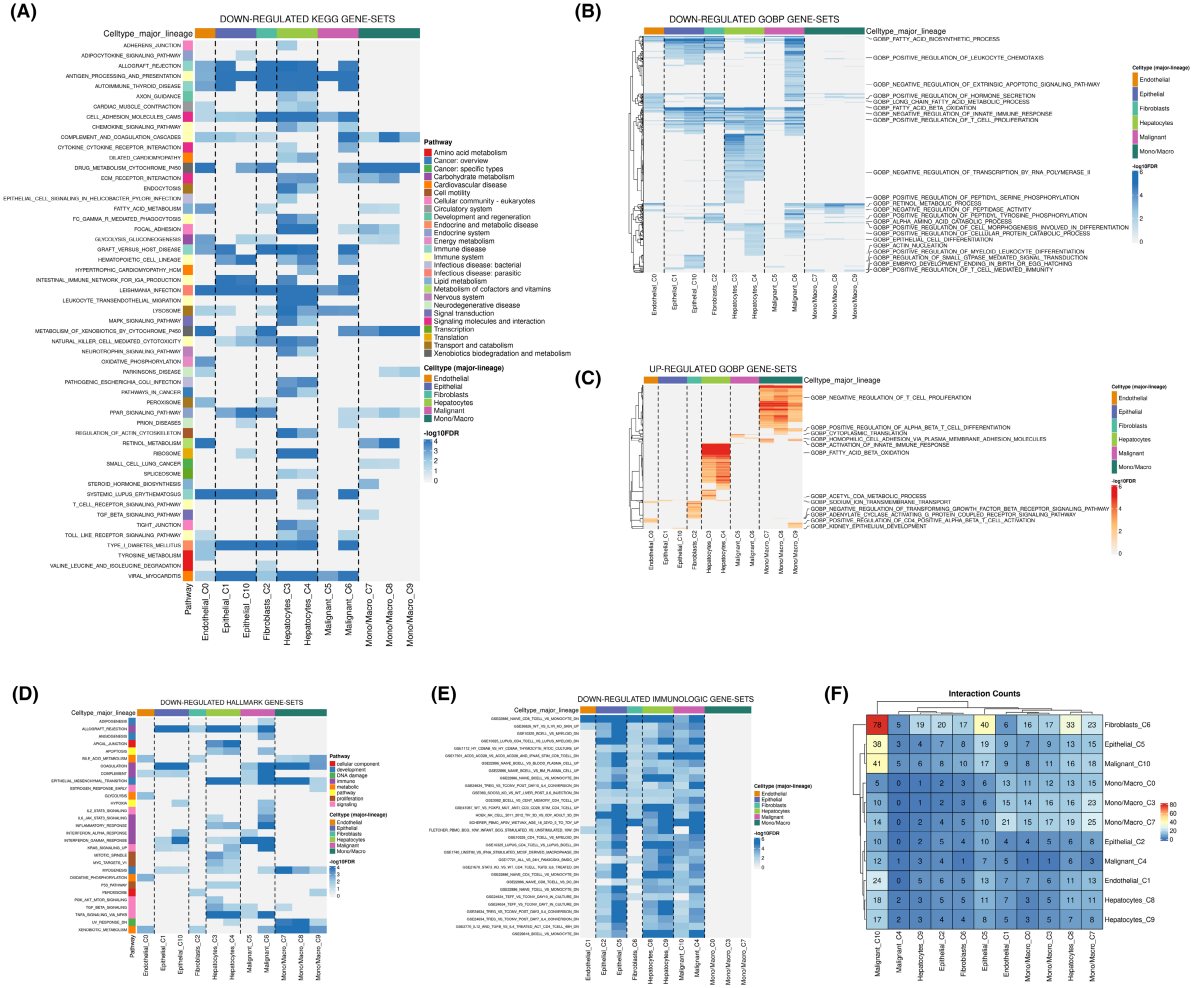
Biological role of endothelial cells in HCC microenvironment. (A) The downregulated KEGG gene sets in which endothelial cells participates (HCC microenvironment). (B) The downregulated GO‐BP gene sets in which endothelial cells participates (HCC microenvironment). (C) The upregulated GO‐BP gene sets in which endothelial cells participates (HCC microenvironment). (D) The downregulated Hallmark gene sets in which endothelial cells participates (HCC microenvironment). (E) The downregulated immunologic gene sets in which endothelial cells participates (HCC microenvironment). (F) The cell interaction of the cells in the HCC microenvironment.

Following the observation of IL33's predominant distribution in endothelial cells, our subsequent analysis delved into the role of endothelial cells within the HCC microenvironment. The KEGG results revealed correlations between endothelial cells and the downregulation of activities associated with allograft rejection, processing and presentation, autoimmune thyroid disease, fatty acid metabolism, glycolysis gluconeogenesis, leishmania infection, P450 metabolism of xenobiotics by cytochrome and retinol metabolism (Figure 2A). GO analysis further unveiled that endothelial cells were associated with the downregulation of activities related to the fatty acid biosynthetic process, fatty acid beta‐oxidation, positive regulation of T cell proliferation, and actin nucleation. Conversely, there was an upregulation of activities in acetyl CoA metabolic process, adenylate cyclase‐activating G protein‐coupled receptor signalling pathway and positive regulation of CD4 positive alpha‐beta T cell activation (Figure 2B,C). The Hallmark analysis of endothelial cells indicated a correlation with the downregulation of activities in bile acid metabolism, complement, glycolysis, oxidative phosphorylation and xenobiotic metabolism (Figure 2D). In terms of immunological gene sets, endothelial cells exhibited a correlation with the downregulated level of naïve CD8^+^ T cell versus monocyte (Figure 2E). Furthermore, cell interaction analysis illustrated that endothelial cells predominantly interact with malignant cells within the HCC microenvironment (Figure 2F).

### 3.3 Clinical role of IL33 in HCC

**FIGURE 3:**
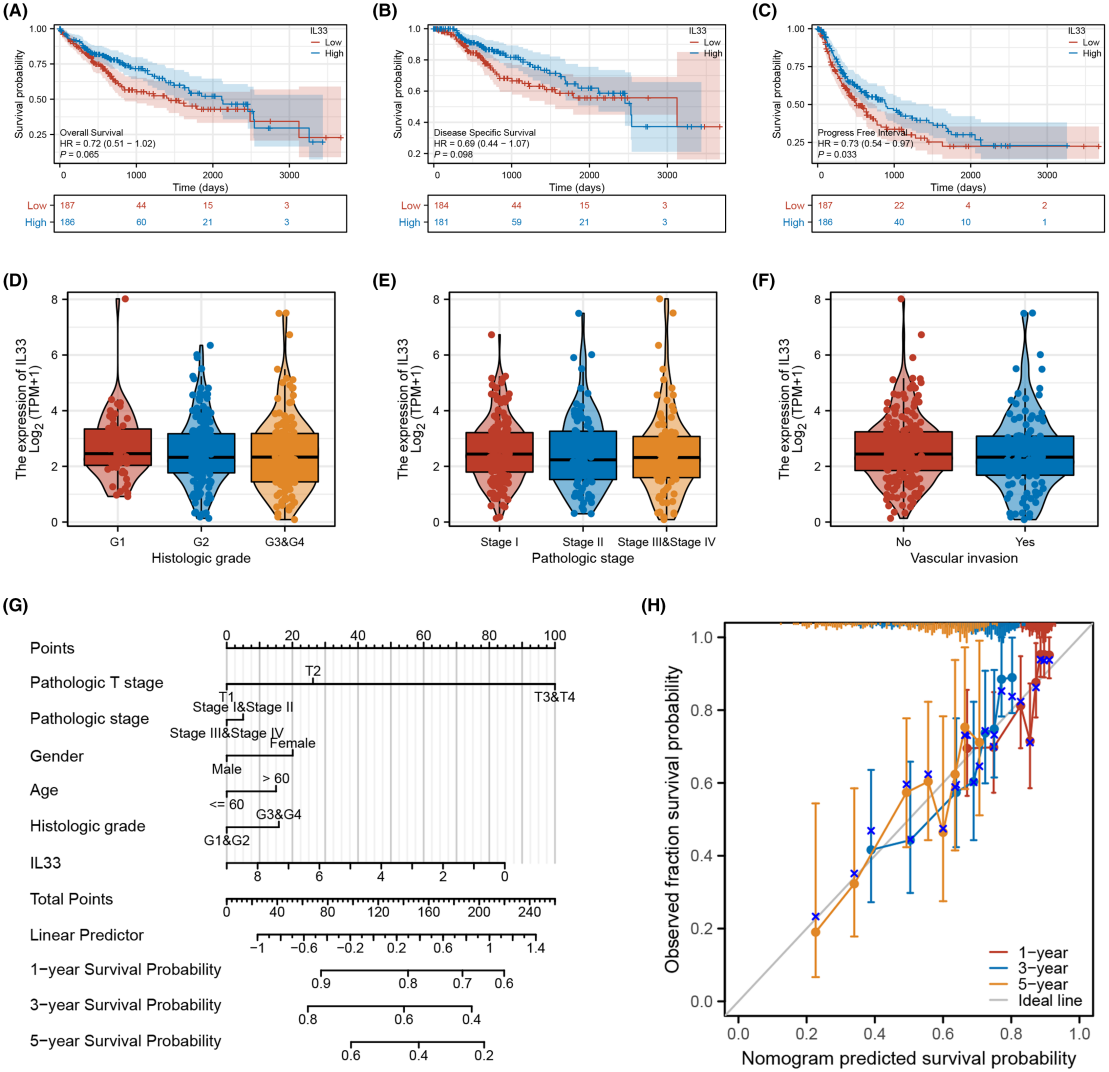
Clinical role of IL33 in the HCC microenvironment. (A) KM survival curves of IL33 in HCC (overall survival). (B) KM survival curves of IL33 in HCC (disease specific survival). (C) KM survival curves of IL33 in HCC (progression free survival). (D–F) The expression level of IL33 in patients with different clinical stage. (G) The nomogram plot combining IL33 level and other clinical features. (H) The calibration curve of the nomogram plot.

Our investigation into the role of IL33 in the HCC microenvironment revealed compelling results. Patients with elevated IL33 expression exhibited a better survival profile, encompassing overall survival, disease‐free survival and progression‐free survival (Figure 3A–C). However, no significant clinical correlations were identified between IL33 expression and certain features such as histologic grade, pathological stage and vascular invasion (Figure 3D–F). To enhance the prognostic predictive capability of IL33 on HCC patient survival, we developed a nomogram plot (Figure 3G). Calibration curves demonstrated that the survival predictions derived from the nomogram plot aligned well with the actual observed survival rates (Figure 3H). These findings underscore the potential prognostic value of IL33 expression in predicting survival outcomes for HCC patients.

### 3.4 Biological enrichment of IL33

**FIGURE 4:**
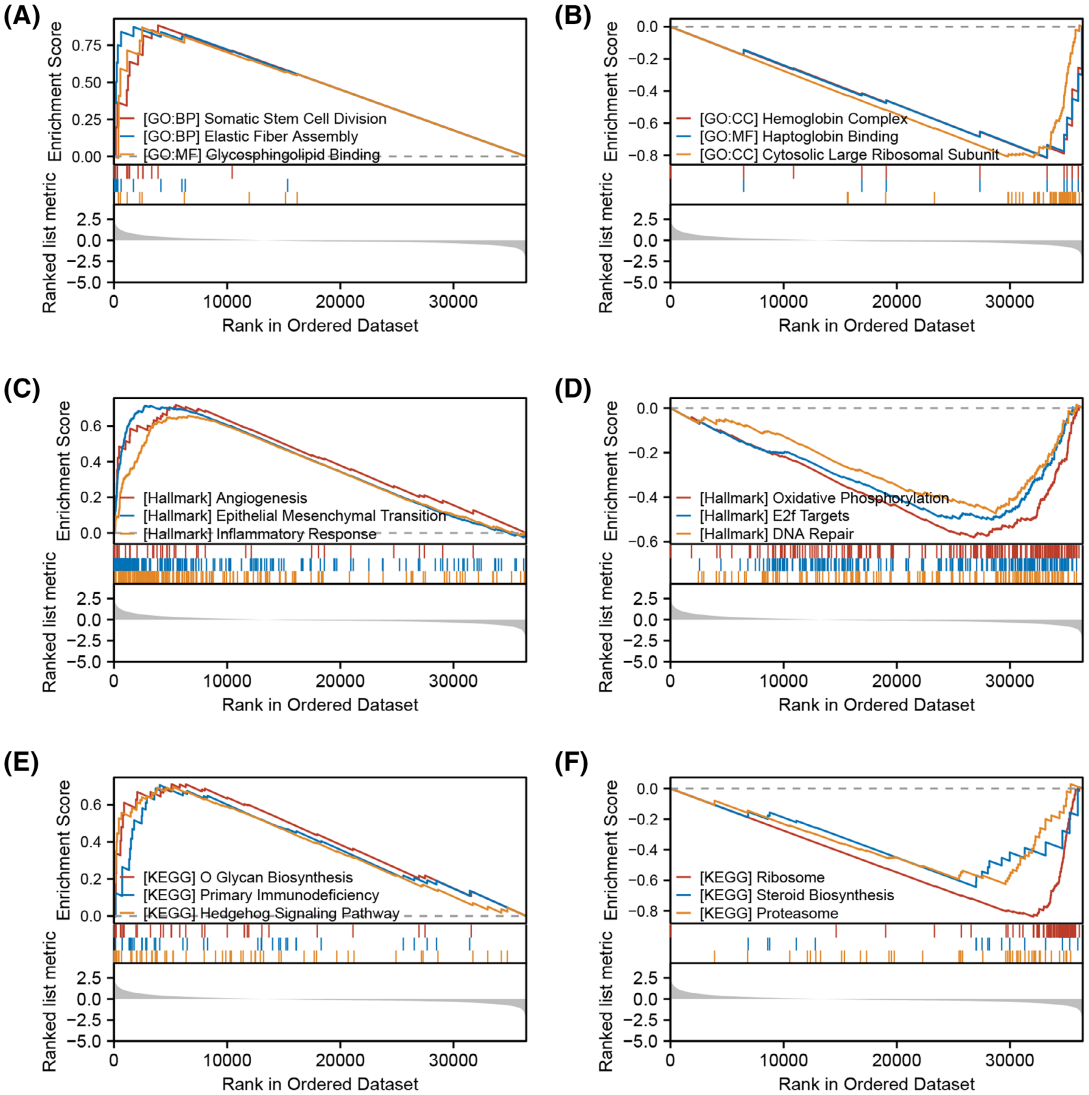
Biological role of IL33 in HCC microenvironment. (A) GSEA analysis of IL33 based on GO gene set (top three upregulated terms). (B) GSEA analysis of IL33 based on GO gene set (top three downregulated terms). (C) GSEA analysis of IL33 based on Hallmark gene set (top three upregulated terms). (D) GSEA analysis of IL33 based on Hallmark gene set (top three downregulated terms). (E) GSEA analysis of IL33 based on KEGG gene set (top three upregulated terms). (F) GSEA analysis of IL33 based on KEGG gene set (top three downregulated terms).

Then, we tried to investigate the underlying biological role of IL33 in the HCC microenvironment. GSEA analysis based on the GO gene set indicated that the top three upregulated terms in the patients with high IL33 expression were somatic stem cell division, elastic fibre assembly and glycosphingolipid binding, while the top three downregulated terms were haemoglobin complex, haptoglobin binding and cytosolic large ribosomal subunit (Figure 4A,B). GSEA analysis based on the Hallmark gene set indicated that the top three upregulated terms in the patients with high IL33 expression were angiogenesis, epithelial‐mesenchymal transition (EMT) and inflammatory response, while the top three downregulated terms were oxidative phosphorylation, E2F targets and DNA repair (Figure 4C,D). GSEA analysis based on the KEGG gene set indicated that the top three upregulated terms in the patients with high IL33 expression were O glucan biosynthesis, primary immunodeficiency and hedgehog signalling pathway, while the top three downregulated terms were ribosome, steroid biosynthesis and proteasome (Figure 4E,F).

### 3.5 Role of IL33 in HCC immune microenvironment

**FIGURE 5:**
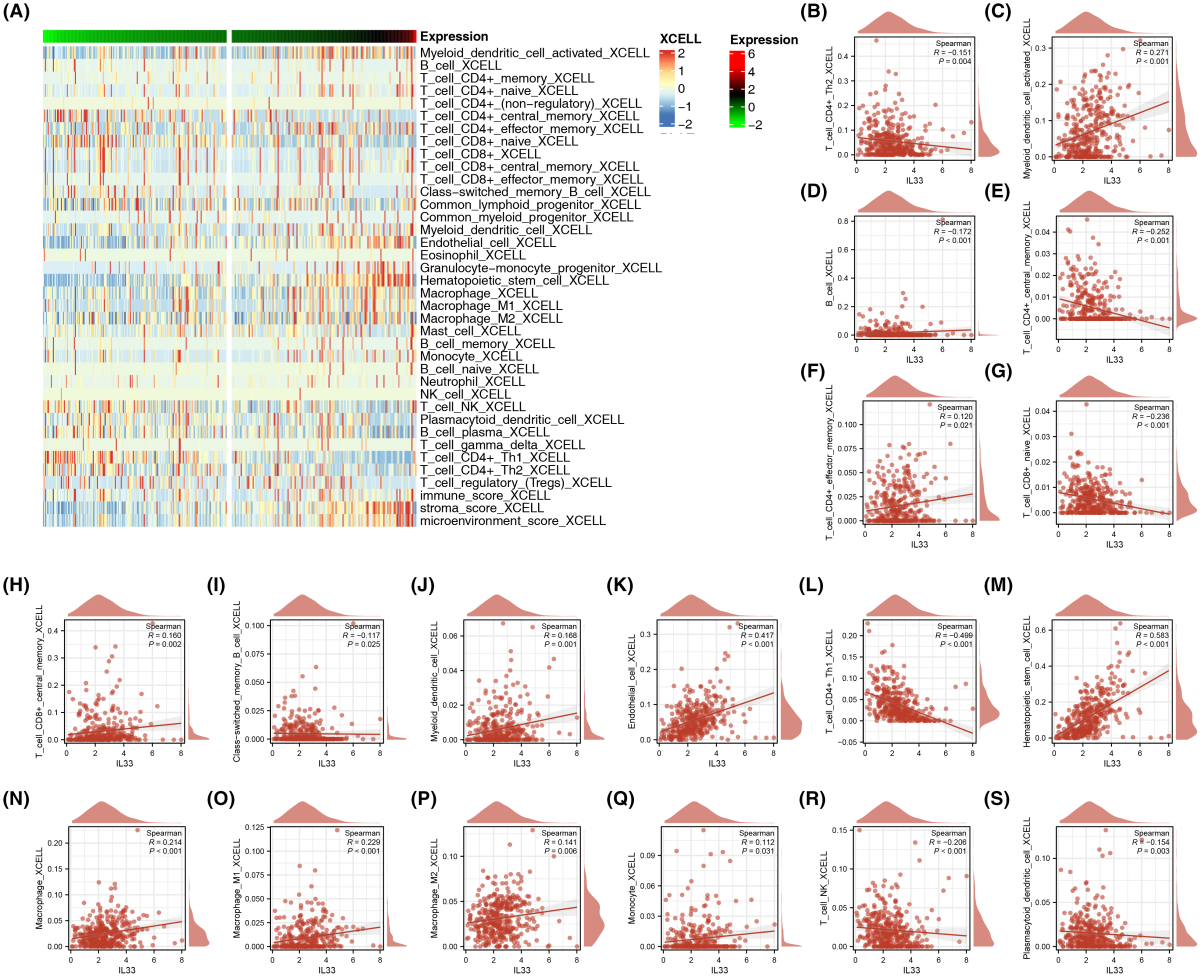
Effect of IL33 on HCC microenvironment. (A) The microenvironment of HCC was quantified by Xcell algorithm. (B–S) Correlation between IL33 and specific immune cells.

Continuing our exploration, we investigated the impact of IL33 in the HCC immune microenvironment. Initially, we utilized the Xcell algorithm to quantify the immune microenvironment of HCC (Figure 5A). The quantified results revealed correlations between IL33 expression and various immune cell types. IL33 exhibited positive correlations with activated myeloid dendritic cells, effector memory CD4^+^ T cells, central memory CD8^+^ T cells, myeloid dendritic cells, endothelial cells, haematopoietic stem cells, macrophages, M1 macrophages, M2 macrophages and monocytes. Conversely, IL33 showed negative correlations with CD4^+^ Th2 T cells, B cells, central memory CD4^+^ T cells, naïve CD8^+^ T cells, CD4^+^ Th1 T cells and NK T cells (Figure 5B–S).

### 3.6 Genomic features, drug sensitivity and pan‐cancer analysis

**FIGURE 6:**
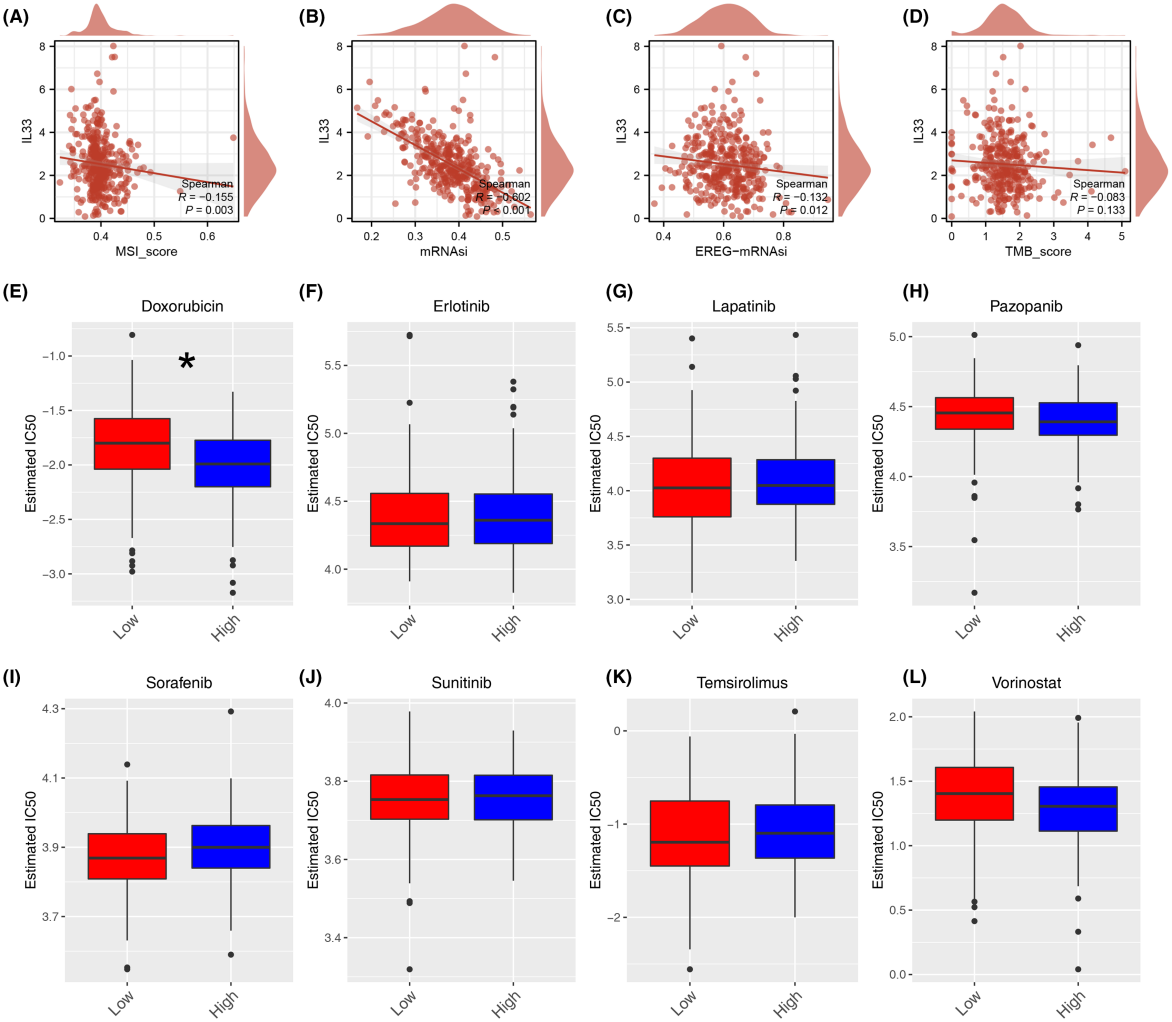
Genomic instability and drug sensitivity. (A) Correlation between IL33 and MSI score. (B) Correlation between IL33 and mRNAsi. (C) Correlation between IL33 and EREG‐mRNAsi. (D) Correlation between IL33 and TMB. (E–L) The IC50 difference in patients with high and low IL33 expression, *p < 0.05.

**FIGURE 7:**
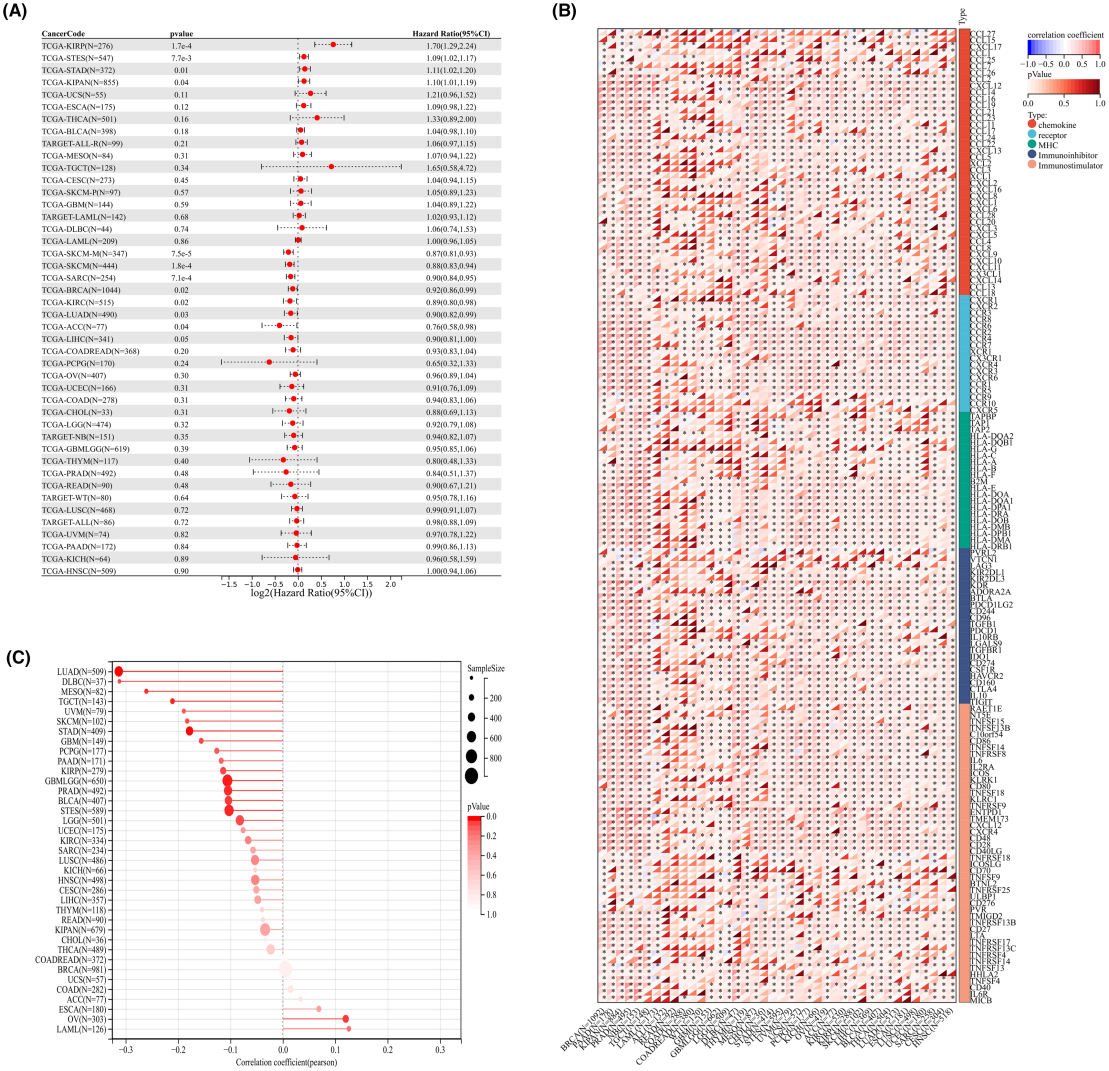
Pan‐cancer analysis of IL33. (A) Univariate Cox regression analysis of IL33 in pan‐cancer. (B) Correlation between IL33 and immune related molecules in pan‐cancer. (C) Correlation between IL33 and TMB score in pan‐cancer.

Simultaneously, our analysis revealed that IL33 exhibited a negative correlation with MSI score, mRNAsi, and EREG‐mRNAsi, although no significant correlation was observed with the TMB score (Figure 6A–D). Additionally, we explored the differences in drug response between patients with high and low IL33 expression. The results demonstrated that patients with elevated IL33 levels showed increased sensitivity to doxorubicin (Figure 6E–L). Furthermore, we sought to elucidate the role of IL33 across various cancers. The findings indicated that in most cancers, IL33 functions as a tumour‐inhibitor molecule (Figure 7A). The correlation between IL33 and immune‐related molecules was illustrated (Figure 7B), along with the correlation between TMB and IL33 in pan‐cancer (Figure 7C).

## 4 DISCUSSION

HCC represents a major global health challenge, characterized by its high incidence and mortality rates.^23^ As one of the most prevalent forms of liver cancer, HCC is often diagnosed at advanced stages, limiting treatment options and contributing to its poor prognosis.^24^ The intricate interplay of molecular and immune factors within the HCC microenvironment underscores the need for targeted therapeutic approaches.^25^ Targeted therapies have emerged as a critical avenue in HCC treatment, aiming to address specific molecular aberrations and signalling pathways implicated in cancer progression. Understanding the molecular landscape and immune interactions in HCC is pivotal for the development of effective targeted interventions, offering promising prospects for improved patient outcomes and personalized treatment strategies.^26^

In our study, we comprehensively explored the role of IL33 in HCC. IL33 exhibited differential expression across various cancer types. Further exploration at the single‐cell level highlighted its primary expression in endothelial cells. The role of endothelial cells within the HCC microenvironment was elucidated, revealing correlations with specific biological processes, gene sets, and immune interactions. Notably, patients with elevated IL33 expression in HCC demonstrated a better survival profile, emphasizing its potential prognostic significance. The developed nomogram plot further enhanced the predictive capability of IL33 on HCC patient survival. Biological enrichment analyses shed light on the intricate biological roles of IL33, revealing associations with stem cell division, angiogenesis and inflammatory response. Moreover, IL33's impact on the HCC immune microenvironment was delineated, showcasing correlations with diverse immune cell types. Genomic features, drug sensitivity analyses and pan‐cancer assessments provided valuable insights into the broader implications of IL33. The negative correlation with MSI score, mRNAsi, and EREG‐mRNAsi, along with increased sensitivity to doxorubicin in patients with high IL33 expression, adds depth to our understanding of its role in cancer therapy.

Some studies have explored the role of IL33 in cancers. For example, Shani et al. discovered that IL33 derived from fibroblasts promotes the metastasis of breast cancer by altering the immune microenvironment and inducing Type 2 immunity.^10^ Alam et al. observed that the mycobiome associated with fungi induces the secretion of IL‐33 and promotes Type 2 immunity in pancreatic cancer.^11^ Fang et al. provided evidence showing that the activation of JNK and recruitment of macrophages by IL33 contribute to the promotion of stemness in colon cancer cells.^27^ Moreover, Kudo‐Saito found that IL33 is a key driver of treatment resistance of cancer.^28^

The result of the single‐cell analysis indicated that IL33 was mainly expressed in the endothelial cell. Endothelial cells play a crucial and complex role in the tumour microenvironment, exerting profound effects on tumour biology and treatment responses.^29^ First, endothelial cells regulate tumour blood supply through angiogenesis, providing essential oxygen and nutrients for tumour growth and dissemination.^30^ This process, known as neovascularization, is vital for tumour survival and development. Second, endothelial cells modulate the tumour cell microenvironment by secreting growth factors, extracellular matrix, and cell adhesion molecules.^31^ These interactions influence tumour proliferation, migration, and invasion, directly shaping the tumour microenvironment. Additionally, endothelial cells play a significant role in immune regulation, impacting the infiltration and activity of immune cells in the tumour microenvironment through changes in surface molecules and cell interactions.^32^ By regulating tumour‐related inflammatory responses, endothelial cells influence tumour immune evasion and the formation of drug resistance. Finally, the role of endothelial cells extends to their impact on tumour treatment.^33^ Their involvement in tumour angiogenesis and drug delivery, as well as their participation in the formation of drug tolerance, makes them a crucial factor in therapeutic strategies.

The elucidation of IL33's biological functions in the HCC microenvironment holds significant implications for its role in cancer pathogenesis. The upregulation of somatic stem cell division, elastic fibre assembly, and glycosphingolipid binding in patients with elevated IL33 expression suggests a potential contribution to cancer stemness, extracellular matrix remodelling, and interactions that facilitate tumour progression and metastasis.^34^ These processes are well‐established hallmarks of cancer development, signifying IL33's involvement in promoting key aspects of malignancy. In the context of the Hallmark gene set analysis, the upregulation of angiogenesis, EMT, and inflammatory response underscores IL33's potential to enhance tumour vascularization, and invasiveness, and create a pro‐inflammatory tumour microenvironment. These factors collectively contribute to the aggressive behaviour of cancer cells, supporting their survival, migration, and evasion of immune surveillance.^35^ Furthermore, the downregulation of oxidative phosphorylation, E2F targets, and DNA repair pathways suggests a potential role of IL33 in suppressing cellular processes associated with maintaining genomic stability. Dysregulation of these pathways is a common feature in cancer, contributing to genomic instability and increased susceptibility to mutations, a hallmark of tumorigenesis.^36^ The KEGG gene set analysis, revealing upregulated terms related to immune responses (primary immunodeficiency) and signalling pathways (hedgehog signalling pathway), suggests that IL33 may modulate the immune microenvironment in HCC. This modulation could impact the interactions between tumour cells and the immune system, influencing immune evasion mechanisms and promoting a tumour‐permissive environment.^37^ In summary, IL33's biological functions in HCC, as indicated by GSEA, point towards its involvement in promoting cancer stemness, affecting tumour progression, modulating the immune microenvironment, and contributing to genomic instability.

The discussion of our findings warrants consideration of certain limitations that may impact the robustness of our conclusions. First, reliance on publicly available data introduces the potential for systematic biases. Public datasets may inherently carry variations, and any systemic biases present in these data could influence the outcomes of our analysis. It is crucial to acknowledge this limitation as it may introduce uncertainties in the generalizability and reliability of our conclusions. Second, while the majority of algorithms employed in our study are stable and rigorous, it is imperative to recognize the inherent limitations posed by the current state of biological knowledge. The field of bioinformatics is dynamic, with continuous updates and refinements. As our understanding of biological processes evolves, the stability of our conclusions may be subject to change. Future advancements in bioinformatics may introduce new insights or alter existing interpretations, emphasizing the need for ongoing scrutiny and validation of our findings. Third, our study employs IC_50_ values from the GDSC database, interpreted as relative, not absolute, measures of drug sensitivity. This approach, while valuable for assessing relative effectiveness across cancer cell lines, deviates from traditional IC_50_ interpretations as strictly positive metrics indicating 50% inhibition concentration. Consequently, our representation of IC_50_ as both negative and positive values for indicating drug sensitivity magnitude introduces a conceptual complexity.

## AUTHOR CONTRIBUTIONS

**Lifang Wei:** Conceptualization (equal); data curation (equal); formal analysis (equal); funding acquisition (equal); methodology (equal); software (equal); writing – original draft (equal). **Ping He:** Conceptualization (equal); data curation (equal); validation (equal); writing – original draft (equal). **Zhongqiu Tan:** Data curation (equal); funding acquisition (equal); investigation (equal); methodology (equal); software (equal); supervision (equal); validation (equal); visualization (equal); writing – original draft (equal). **Cheng Lin:** Conceptualization (equal); funding acquisition (equal); methodology (equal); resources (equal); software (equal); validation (equal); visualization (equal). **Zhongheng Wei:** Conceptualization (equal); data curation (equal); formal analysis (equal); investigation (equal); supervision (equal); validation (equal).

## FUNDING INFORMATION

This work was supported by the National Nature Science Foundation of China (NO: 82260532), and the Scientific Project of High Talent of Affiliated Hospital of Youjiang Medical University for Nationalities (NO: R20196327).

## CONFLICT OF INTEREST STATEMENT

The authors confirm that there are no conflicts of interest.

## Data Availability

Data can be obtained from the corresponding author based on the reasonable request.
